# Analysing and recommending options for maintaining universal coverage with long-lasting insecticidal nets: the case of Tanzania in 2011

**DOI:** 10.1186/1475-2875-12-150

**Published:** 2013-05-04

**Authors:** Hannah M Koenker, Joshua O Yukich, Alex Mkindi, Renata Mandike, Nick Brown, Albert Kilian, Christian Lengeler

**Affiliations:** 1 Johns Hopkins Bloomberg School of Public Health Center for Communication Programs, Baltimore, MD, USA; 2 Tulane University, New Orleans, LA, USA; 3 Booz Allen Hamilton, Dar es Salaam, Tanzania; 4 National Malaria Control Programme, Ministry of Health and Social Welfare, Dar es Salaam, Tanzania; 5 Swiss Tropical and Public Health Institute, Basel, Switzerland; 6 A To Z Textile Mills Ltd, Arusha, Tanzania; 7 Tropical Health LLP, Montagut, Spain; 8 University of Basel, Basel, Switzerland

**Keywords:** Long-lasting insecticidal nets, Malaria, Schools, Continuous distribution, Universal coverage, Tanzania, Insecticide-treated nets, Mass campaign

## Abstract

**Background:**

Tanzania achieved universal coverage with long-lasting insecticidal nets (LLINs) in October 2011, after three years of free mass net distribution campaigns and is now faced with the challenge of maintaining high coverage as nets wear out and the population grows. A process of exploring options for a continuous or “Keep-Up” distribution system was initiated in early 2011. This paper presents for the first time a comprehensive national process to review the major considerations, findings and recommendations for the implementation of a new strategy.

**Methods:**

Stakeholder meetings and site visits were conducted in five locations in Tanzania to garner stakeholder input on the proposed distribution systems. Coverage levels for LLINs and their decline over time were modelled using NetCALC software, taking realistic net decay rates, current demographic profiles and other relevant parameters into consideration. Costs of the different distribution systems were estimated using local data.

**Results:**

LLIN delivery was considered via mass campaigns, Antenatal Care-Expanded Programme on Immunization (ANC/EPI), community-based distribution, schools, the commercial sector and different combinations of the above. Most approaches appeared unlikely to maintain universal coverage when used alone. Mass campaigns, even when combined with a continuation of the Tanzania National Voucher Scheme (TNVS), would produce large temporal fluctuations in coverage levels; over 10 years this strategy would require 63.3 million LLINs and a total cost of $444 million USD. Community mechanisms, while able to deliver the required numbers of LLINs, would require a massive scale-up in monitoring, evaluation and supervision systems to ensure accurate application of identification criteria at the community level. School-based approaches combined with the existing TNVS would reach most Tanzanian households and deliver 65.4 million LLINs over 10 years at a total cost of $449 million USD and ensure continuous coverage. The cost of each strategy was largely driven by the number of LLINs delivered.

**Conclusions:**

The most cost-efficient strategy to maintain universal coverage is one that best optimizes the numbers of LLINs needed over time. A school-based approach using vouchers targeting all students in Standards 1, 3, 5, 7 and Forms 1 and 2 in combination with the TNVS appears to meet best the criteria of effectiveness, equity and efficiency.

## Background

Between 2009 and 2011 Tanzania implemented two mass long-lasting insecticide-treated bed net (LLIN) distribution campaigns with the goal of achieving universal coverage nationwide: the under-five catch up campaign (U5CC) and the universal coverage campaign (UCC). Approximately 27 million LLINs were distributed during the two campaigns, leading to large increases in household ownership and usage [1]. Reaching universal coverage through campaigns represents a “catch-up” strategy according to the Consensus Statement of the Roll Back Malaria Vector Control Working Group (VCWG) [2]. However, it is well recognized that once countries reach universal coverage they need to implement new strategies to maintain the high coverage levels: “Keep-Up” strategies. New households require new nets and lost or worn out nets need to be replaced.

Tanzania introduced the Tanzania National Voucher Scheme (TNVS) in 2004 to distribute nets to pregnant women and infants. During the period of the recent campaigns the TNVS distributed roughly 5.4 million nets. The TNVS has been extensively described in terms of operations and cost-effectiveness [3-10]. Vouchers obtained by pregnant women and mothers of infants coming for measles vaccination through routine clinic visits can be redeemed for reduced price nets at participating retailers. Top-ups paid by women and mothers initially ranged from about TZS700 to over TZS1, 500 ($0.65-$1.38 at 2005 rates) depending on the size of the selected net [11]. In late 2006 the voucher value was increased to TZS3, 250 ($2.60 at 2006 rates) and the infant voucher was introduced. By 2008, the average top-up had reached over TZS2, 300 ($1.95 at 2008 rates) [12]. In October 2009 the existing voucher was replaced by a new voucher with a fixed top-up of TZS500 ($0.38 in 2009 and $0.32 in 2012). However, the TNVS does not provide enough nets annually to maintain universal coverage, hence the need for defining and implementing additional “Keep-Up” strategies. This paper describes the results of a comprehensive process of stakeholder meetings, in-depth interviews and cost and coverage modelling to identify options for maintaining universal coverage in Tanzania. This work was carried out also considering that the current funding for malaria control at global level is plateauing and potentially declining [13]; hence new strategies must be both effective and efficient.

## Methods

### Qualitative methods

A series of bilateral and multilateral stakeholder meetings was held in Dar es Salaam, Morogoro, Mtwara, Mwanza and Arusha, reflecting a variety of transmission zones and combinations of malaria control interventions. Field visits and in-depth interviews were conducted in each zone with participating TNVS clinics and retailers along with local government officials, council health management teams, district medical officers and malaria focal persons. Stakeholder meetings were designed to elicit inputs on the operational feasibility of each of the options for additional channels and to identify potential bottlenecks and barriers at both government and community level to successful implementation. Interviews with retailers and net manufacturers focused on retailers’ experience under the TNVS and their current business practices. These meetings took place between 1 and 24 June, 2011. Additional file 1 describes the details of stakeholder meeting attendees, interviewees and site visits.

### Modelling and costing methods

The term “coverage” is defined here as it is used within the National Malaria Control Programme of Tanzania (NMCP), to mean use of nets by the general population. “Ownership” is defined as household ownership of a specified number of nets per household, or net to occupant ratio. Unless otherwise noted, this is the standard Roll Back Malaria (RBM) indicator of at least one LLIN per household.

**Figure 1:**
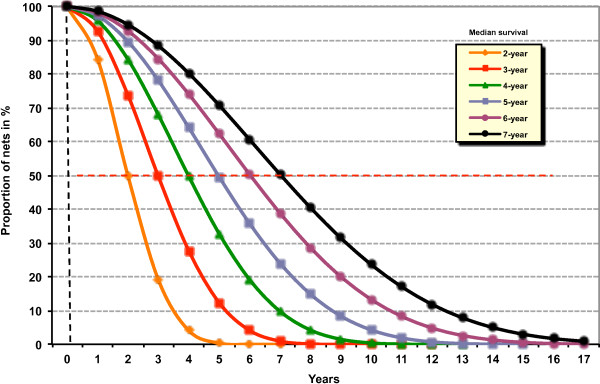
Decay curves used to model net longevity in NetCALC (Kilian).

Net ownership and coverage levels were modelled using the NetCALC software developed on a Microsoft Excel platform (available at http://networksmalaria.org). NetCALC projects LLIN coverage at a given point in time as a simple compartmental model based on the number of nets available for use at that time which in turn is the sum of all nets remaining from annual net cohorts distributed assuming an S-shaped loss-function (Figure 1). This loss function is built on data and observations on the longevity of LLINs in the field [14-16] which was then mathematically described, allowing the user to select a variety of median survival times with the default set at three years. The relationship between the mean number of nets per net-owning household and the mean number of nets per household considering all households was defined by relating the range of coverage rates from several household surveys in Uganda and the mean number of nets per net-owning households and calculating the corresponding ratio of nets to households. Plotting the results showed a curvilinear function. This relationship was modelled in STATA using a fractional polynomial regression (the fracpoly command) and a correction factor was added to account for variations in average household size from country to country. Thus NetCALC is able to translate the remaining number of nets into a value for household net coverage (expressed as the percentage of households owning at least one LLIN) via this formula. The correction factor in the formula also ensures that 100% household coverage with at least one LLIN is equivalent to the population divided by 1.8 [15], which is the World Health Organization (WHO) and RBM recommendation for the number of insecticide-treated nets (ITNs) needed for universal coverage (WHO 2011). Hence, by inputting population parameters, the expected mean lifespan of local LLINs, and numbers of LLINs distributed, NetCALC estimates ownership (at least one LLIN per household) over a set time period.

Models were implemented using NetCALC’s Free Modeling module to allow for complete control over the numbers of nets assumed to be delivered in the “Keep-Up” system. The numbers of nets delivered using the continuous supply systems of the TNVS were estimated utilizing the continuous Antenatal Care-Expanded Programme on Immunization (ANC/EPI) modules. Base scenarios utilized a mean lifespan of three years for all LLINs distributed (all nets distributed in the U5CC and UCC campaigns and since October 2009 under TNVS have been Olyset™ polyethylene nets produced by A to Z Textile Mills Ltd in Arusha [17]). Details of model inputs and outputs are included as a web appendix.

### Population structure and population protected

The population structure used in the model is based on that of Tanzania with an estimated population of 43.2 million in 2010, 17% of whom are children under five and 47.4% under 15 years of age based on the Tanzania National Bureau of Statistics (NBS) projections from the 2002 Census. The average household size was assumed to be five persons and this was treated as invariant over time [18]. A population growth rate of 3.0% per annum was assumed [19]. School enrolment figures were derived from Tanzanian National Enrolment Management Information System Statistics [20]. Other inputs were estimated based on the 2010 Tanzania Demographic and Health Survey [18].

### Time frame

Inputs of LLINs were estimated over the period 2009-2021 inclusively, however, only additional nets beyond the mass campaigns and TNVS deliveries through 2011 were included in the analysis of cost, LLIN needs and cost-effectiveness. In order to accurately estimate current coverage levels and net ages nationwide it was necessary to include nets that had been delivered before the decision point of June 2011. However, nets that had either already been delivered or were planned for delivery were considered as “sunk-costs” and not included in the decision analysis.

### Net inputs

Numbers of LLINs distributed in Tanzania through the TNVS were assumed to increase only in proportion to population growth, until the end of 2011. Inputs of LLINs during the U5CC Campaign and a completed UCC were estimated based on information from the Tanzania NMCP and included in the model in the year of actual (or planned) distribution [1,17]. TNVS inputs were estimated based on the assumption of 95% coverage of ANC and first EPI vaccinations [18], 90% success at vouchers being received by the target groups and 80% redemption rate based on current data from the Mennonite Economic Development Associates (MEDA), the non-governmental organization implementing the voucher distribution and redemption component of the TNVS (Faith Patrick, pers comm, June 2011). Expansion of the voucher system beyond the current year assumed 90% of target groups would receive a voucher and that 80% would redeem them, consistent with estimated voucher redemption rates, but higher than historical redemption rates when the “top-up” associated with voucher redemption was higher in real and nominal terms than currently. Based on previous campaign success in reaching targeted households, direct delivery of nets through campaigns or other distribution assumed that 80% of targeted groups would receive nets [1,17,21-28].

### LLIN needs

The Tanzania NMCP has established a target of 80% LLIN use within the general population. To account for a probable small but persistent gap between ownership and use over the 10-year period, stakeholders estimated at the first stakeholder meeting in Dar es Salaam that a 7% disparity could serve as a standard adjustment for non-use. This was included in the modelling so that outputs would indicate whether or not Tanzania would meet its target of 80% LLIN use. In order to maintain coverage at this level the minimum number of additional LLIN per year from 2012 to 2021 was estimated. The coverage target was calculated based on the total number of person-years in Tanzania over the period 2012 to 2021 and the proportion of all person-years over the same period in which a net was used. Success at meeting the NMCP target resulted when at least 80% of all possible person-years over the period were protected.

### Cost and cost-effectiveness

Cost modelling used data from various sources, including the NMCP of Tanzania, published literature, and financial records and reports of implementing partners of the NMCP. Conversions to USD from TZS were made using an exchange rate for 2011 (TZS1,500 per USD), the prevailing exchange rate at the time of the study. All costs were projected into the future assuming constant dollars and adjusted to present value in 2012 using a 3% discount rate as is common in the economic evaluation literature. All cost analyses took a provider perspective and costs were purely financial. Only costs related to actual financial outlays by donor agencies or the NMCP of Tanzania were included. Commodity costs for nets were assumed not to vary over time, but were related to scale in the mass distribution campaign models (where the largest procurements would occur), so that larger purchase quantities of nets resulted in lower per-net prices. The relationship between purchase volume and LLIN procurement cost was estimated based on LLIN tender data from past procurements in Tanzania. In commercial sales scenarios, sales levels were estimated based on historical sales records from the Strategic Social Marketing for expanding the Commercial Market of ITNs in Tanzania (SMARTNET) programme adjusted for population size and subsidy levels using existing price elasticity estimates [10]. Costs to users were not included in total costs as only purely provider costs were considered in the cost analysis. The implications of varied user costs were included in the sense that various levels of voucher redemption and sales volumes related to top-ups and market price were considered.

The costs of voucher distribution were assumed to be similar to the current TNVS. Direct net distribution assumed the same costs as for past campaign distributions. Other than the purchase quantity efficiencies assumed in the mass distribution models, economies of scale were not included. The evidence of scale efficiencies in LLIN distribution is limited and no data to base such a model on currently exists [29]. Similarly, no economies of scope were assumed in the modelling of combination approaches. In costing the options, campaign or physical net distribution costs included campaign-specific behaviour change communication (BCC) activities and follow up “hang-up” activities. However, no unlinked BCC activities were included either for campaigns or any other distribution system. Denominators for cost-effectiveness ratios were estimated in terms of the total person-years covered over the period 2012-2021.

### Sensitivity analysis

One-way sensitivity analyses were conducted on net lifetimes, costs of delivery systems and net procurement prices, usage rates and discount rates. Multiway or scenario analyses were also conducted to estimate the effects of varying multiple parameters on the number of LLINs needed, total costs and cost-effectiveness.

## Results

### Numbers of LLINs required for meeting universal coverage targets in Tanzania

**Figure 2:**
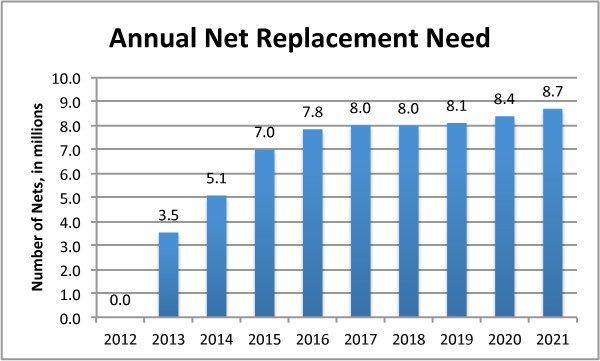
Annual net replacement need for Tanzania, 2012-2021.

Under the base scenario an average of 7.9 million LLINs per year (2012-2021) were required to meet NMCP targets of maintaining 80% coverage (Figure 2). Relatively few nets were needed in the first years (2012-2013) due to the large numbers of nets already present from recent mass campaigns. After 2013, however, there was a need for a significant increase in scale. Sensitivity analysis indicated that both the size and timing of the increase in delivery was highly dependent on the assumed lifetime of nets delivered in the two mass campaigns and to a lesser extent the number of LLINs delivered through the TNVS. Assuming a constant lifetime of nets over the period, the number of LLINs needed in each year after the programme’s initial expansion grew in direct proportion to population growth.

### Stakeholder preferences for “Keep-Up” strategies

An ideal “Keep-Up” strategy, as defined through stakeholder meetings and in consultation with the NMCP, would maintain usage of LLINs at 80% or more of the general population of Tanzania and be equitable in terms of access to LLINs. It would additionally have minimal geographic and temporal gaps in coverage, so that spatial coverage is maintained over time throughout all communities, in order to provide the significant community effects (which have been measured at >50% household coverage in Kenya [30] and shown to exceed individual protection at levels over 40% in a mathematical model [31]). Further, the system should not oversupply nets to households or be excessively costly and burdensome to manage and administer. It would also put some degree of responsibility on households to acquire nets, either through effort (travel, self-registration, et.) or through paying a small portion of the cost of the net as is currently done through the TNVS. The system would also encourage fair competition among manufacturers to improve quality and reduce costs. In an ideal system a choice of nets (in terms of size, fabric and colour) would also be available to the consumer.

At all the stakeholder meetings, priority groups were identified fairly consistently, and included pregnant women, under-fives, the elderly (over 60), the disabled, orphans and street children, people living with HIV/AIDS and tuberculosis, the poor, widows and widowers, and victims of floods/fire and other disasters.

Possible net distribution options are described systematically in the following sections, first by presenting the results of stakeholder discussions, and then by modelling number of nets and cost of the approach. Single interventions are examined first, and the most attractive combinations follow.

### Single interventions

Repeated mass campaigns, the TNVS alone, health-facility distribution of nets, community distribution of nets or vouchers, school-based distribution of nets or vouchers, and commercial sector options were analysed individually first as future options for the “Keep-Up” strategy. Additional file 2 summarizes these options.

#### Future option 1: repeated mass campaigns

Tanzania has conducted two mass LLIN distribution campaigns: the 2009-2010 U5CC and the 2010-2011 UCC). Completed in November 2011, these campaigns distributed 27 million nets with an estimated financial cost of $7.07 per net for the U5CC [17] and $5.90 per net for the UCC [1]. Over a 10-year period around 61.5 million nets would be needed to conduct a UCC every three years, accounting for population growth. Given the scale and complexity of implementation, any future UCC is likely to be conducted in rolling stages with specific regions or zones covered sequentially – as was done for the 2010-2011 campaign. While in the national aggregate this strategy would provide protection to approximately 80% of all of the person-years at risk over 2012-2021, the fluctuation in coverage would be fairly large in individual locations, with predicted LLIN use at any location varying from 93% of the population at peak periods (just after completion of distribution) to as low as 20% in periods between campaigns.

**Figure 3:**
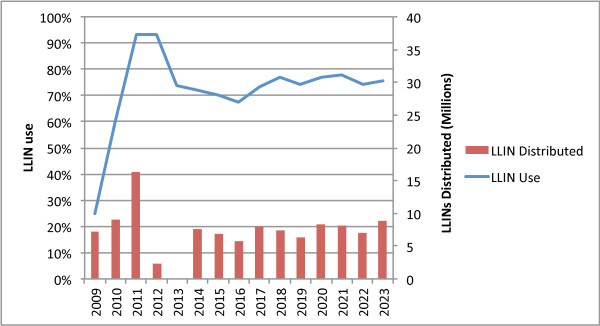
Rolling Universal Coverage Campaign every three years - National Coverage.

**Figure 4:**
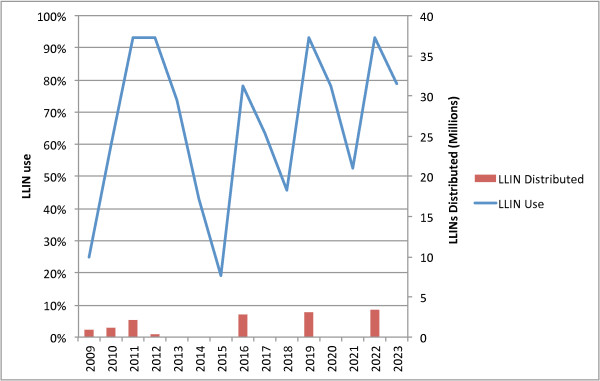
**Rolling Universal Coverage Campaign every three years - Southern Zone.** Southern Zone is last to receive a second campaign under the model, thus the decline in coverage is most extreme in this area.

Figure 3 illustrates modelled results of conducting a rolling universal coverage campaign, segmenting Tanzania into six zones each with a campaign every three years on a six-month rotation schedule (campaign sizes are adjusted for population growth). Figure 4 illustrates modelled results of coverage for Southern Zone, which was one of the first zones to complete universal coverage, but (in this model) one of the last to receive the second universal coverage distribution, resulting in a significant loss of coverage between distributions.

#### Future option 2: Tanzania national voucher scheme (TNVS)

At current levels, the TNVS distributes about 1.5-1.6 million nets per year. MEDA projections assume that 90% of pregnant women and infants attend ANC and EPI and that 85% of pregnant women (80% for the infant voucher) then redeem their respective vouchers, putting the potential throughput at 2.64 million nets per year starting in 2012 (MEDA, pers comm). The network of retailers is currently quantified at 5,426 retailers (confirmed as of June 2011). A total of 4,428 out of a possible 4,891 Reproductive and Child Health (RCH) clinics participate, including some private clinics [32]. By continuing the TNVS over the next several years coverage can be maintained at high levels at least until the end of 2013, when additional channels would need to be added in order for coverage not to drop below acceptable levels (by 2014 ownership would fall below 70%).

Interviews with retailers indicated that tying up large amounts of working capital with slow-moving stocks of TNVS nets has been difficult. If turnover were faster the small TZS500 profit from top-up payment by women could become more attractive. The financial and opportunity costs of stocking LLINs were described by retailers, who noted that consumer goods such as soap or food items take up less shelf space and sell in greater volume, bringing in greater profits than bulky LLINs. However, retailers also reported that they felt they were “providing a service” to pregnant women and mothers of young children by stocking nets and that they enjoyed this aspect of the programme. The majority of those interviewed were selling many other products in their shops; LLINs were only a small part of their total sales.

The model used the assumptions that vouchers reach 90% of the beneficiaries attending clinics and that 80% of vouchers are redeemed, based on 2010 Demographic and Health Survey (DHS) findings that 96% of women attend ANC at least once and measles coverage is 85%, and on MEDA’s voucher redemption records from early 2011. Coverage models based on these rates indicate that TNVS by itself would maintain national coverage with LLINs at around 25%.

**Figure 5:**
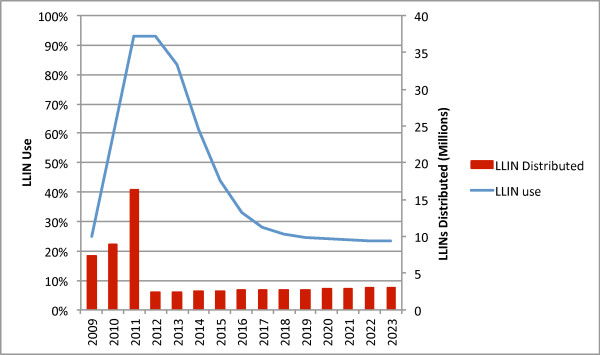
TNVS maintained alone.

Figure 5 describes coverage achieved by maintaining the TNVS in the absence of other distribution channels. Over 10 years this option would require 26.5 million LLINs at a total cost of $182 million, and would provide 45% of total person-years of protection, at a cost per person-year protected of $0.99.

#### Future option 3: ante-natal care (ANC) and expanded programme for immunization (EPI) delivery of nets

A third possibility for delivery of nets to pregnant women and infants would be to distribute free nets directly to beneficiaries through RCH/EPI clinics. Assumptions in the model kept 90% attendance rate at RCH clinics, and assumed that 80% of those attending will receive a net, as LLINs are more difficult to transport and store than vouchers. While this slightly increased coverage in the model, by eliminating the redemption gap, the overall gains in ownership and use appear to be modest. Over 10 years this option would require 29.5 million LLINs at a total cost of $212 million, and would provide 48% of total person-years of protection, at a cost per person-year protected of $1.10.

#### Future option 4: community vouchers or nets

With a ‘community voucher’, community members would identify households in need of replacement nets and issue them vouchers to be redeemed at participating retailers. This approach could in principle deliver the number of LLINs per year set by the programme at any desired level. The system would need to be capped at the number needed to fulfill all replacement needs with some allowance for loss during and immediately after delivery. Community delivery of nets or vouchers was initially a popular option during discussions, as both mechanisms could potentially fill the gaps needed to replace nets and maintain universal coverage. However, a number of potentially significant challenges were cited during discussions, including the difficulty of verifying household need on a regular basis, costs of motivating community volunteers to conduct household outreach to assess need, concerns about favouritism for certain households over others (particularly related to hard-to-reach areas, or based on political affiliations). While stakeholders suggested trading in old nets for new nets as an option to verify household need, they also concluded that this would result in rewarding households that did not take good care of their LLINs. Making vouchers available to the general population at health clinic level was also discussed, and determined to be less desirable again due to difficulties of verifying household need and due to the additional burden that would be placed on health care workers. Conducting regular household registration to identify sleeping spaces that lacked nets was a third option. However, none of the stakeholders thought that this household registration could be accomplished on a volunteer basis; compensating the efforts of local officials or community volunteers who help identify household needs would become significant at scale.

**Figure 6:**
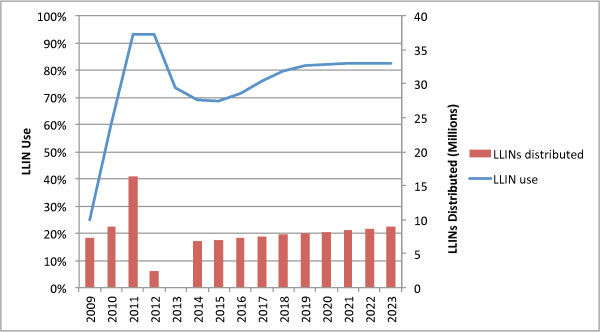
Full-scale Community Voucher.

The model (Figure 6) assumed that community vouchers cover 90% of the need for full replacement of nets and that there would be an 80% redemption rate. Community delivery of nets assumed that 80% of households would receive nets. Over 10 years this option would each require 62.7 million LLINs at a total cost of $477 million (for a community voucher programme) or $432 million (if free net distribution was used), and would provide 78% of total person-years of protection, at a cost per person-year protected of $1.52 (vouchers) or $1.39 (free nets).

#### Future option 5: school vouchers or direct school net delivery

Distribution of vouchers or free LLINs through school registration is another interesting option as a component of a strategy to maintain universal coverage. Enrolment at the primary school level in Tanzania is generally high, with all but two regions reporting net enrolment rates greater than 90% [20]. Additionally, roughly 30% of the population of the country is between five and 15 years of age and is thus eligible for primary school enrolment and at least 62% of households have a current resident of primary school age [18]. This strategy would not reach households who have no school-age children or households that cannot or do not send their children to school, unless there is inter-household redistribution of LLINs (for which there is no evidence to date). Enrolment is the highest for Standard 1 and falls in each subsequent year as students drop out of the school-attending population. Primary school enrolment rates remain much higher than secondary school and thus would likely be the primary target of any net distribution strategy. Results of stakeholder discussions confirmed that beneficiary identification is clear and simple as only students enrolled in school would be eligible and teachers and school health officials could facilitate distribution and monitoring. Stakeholders noted that since households already need to pay for school fees and uniforms at the beginning of the school year, the additional expense of a voucher could pose a barrier to school enrolment, and might be expected to reduce voucher redemption rates. On the other hand, receipt of vouchers could alternatively incentivize enrolment and thus potentially boost enrolment levels.

In Tanzania, as of 2010, there were approximately 8.4 million primary school enrollees. Delivery of vouchers to 90% of all enrollees (with an 80% redemption rate) would lead to the delivery of approximately 6.2 million LLINs per year into households in Tanzania, falling short of total net replacement need. However, this approach might also be used to complement other strategies including the ongoing TNVS, or a community level distribution mechanism (discussed below under the heading of “combinations”). Modelling the delivery via vouchers to all primary school students each year would require 56.7 million LLINs at a total cost of $389 million, resulting in 73% of all person-years protected with a cost of $1.32 per person-year-protected. If free nets were provided instead, due to slight gains in delivery success, 67.3 million LLINs would be required at a total cost of $466 million, resulting in 83% of all person-years protected at a cost of $1.41 per person-year protected (Additional file 2).

#### Future option 6: commercial sector distribution

Very little information exists publicly which can be used to predict the demand for population-wide subsidized sales of LLINs in Tanzania. However, the history of the SMARTNET programme (2002-2007) provides clear evidence that sales could reach more than 1.5 million nets annually with a net price of TZS3,000-5,000 or roughly $2.63 to $4.39 (unpublished data, Population Services International/Tanzania (PSI)). Additionally, a recent study estimated the demand for the pregnant woman voucher and infant voucher redemptions for LLINs at various top-up prices for different socio-economic quintiles [10]. The information from these two sources was used to estimate demand for LLINs on the commercial market at various subsidized retail prices. The accuracy of results modelled here is highly speculative due to the limited evidence base on both general population price elasticity of demand and historical sales volumes for similar (LLIN) products.

**Figure 7:**
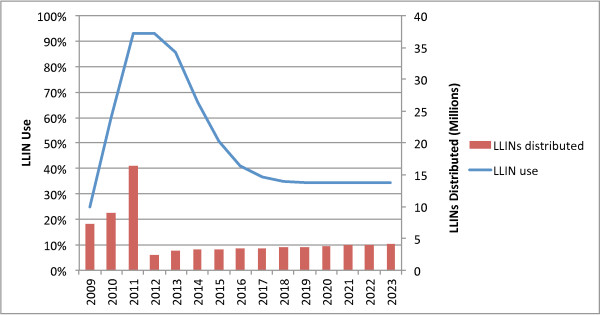
Commercial sector subsidized sales at TZS500 per net.

Figure 7 illustrates the model results of making LLINs available through the retail network to the general population at a highly subsidized retail price of TZS500 ($0.32). Costs of such an approach were modelled based on the current mechanism for providing subsidies to beneficiaries per net sold through the TNVS. At various retail prices more substantial amounts of the manufacture and distribution costs have been assumed to be passed along through retailers to manufacturers and thus subsidy amounts are assumed to be lower at higher top-up prices. (For the results of the extended analysis see accompanying spreadsheet models). This model in its base scenario estimates that approximately three million LLINs could be delivered each year via this mechanism, or roughly 38% of expected replacement need. Over 10 years this option would require 33.4 million LLINs at a total cost of $214 million, and would provide 52% of total person-years of protection, at a cost per person-year protected of $1.00.

### Combinations of options

To model combinations of different mechanisms, overall net inputs from individual channels were combined to estimate final coverage levels. Some delivery systems were modelled on alternative scales in order to avoid oversupplying nets. There was strong support among stakeholders to maintain the TNVS and to combine it with an additional channel. Given current experience with the Medical Stores Department (MSD) supply chain, stakeholders generally agreed that switching from the voucher scheme to a direct delivery of nets via ANC and EPI would likely result in frequent net stock outs, reducing the success of this strategy. Therefore, three potential combinations are modeled here that each include maintaining the TNVS: TNVS plus rolling mass distribution campaigns; TNVS plus school-based distribution; and TNVS plus school- and community-based distribution. These options are summarized in Additional file 2. At this stage, the TNVS represents a working model for a “Keep-Up” mechanism, with a high level of recognition and acceptance, and it would be highly risky not to include this strategy in future plans.

#### Combination 1: TNVS plus rolling mass campaigns

**Figure 8:**
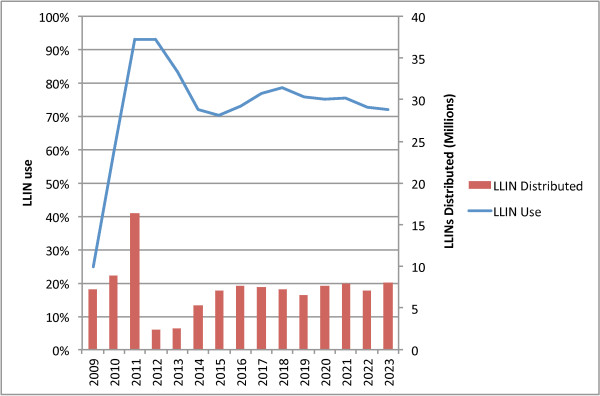
National coverage for TNVS plus three-year rolling UCC.

The combination of the TNVS with repeated mass campaigns for universal coverage on a three- or five-year cycle (assuming a three-year net lifespan) yields universal coverage periodically, but the number of nets delivered by the TNVS fails to supply enough LLINs to ensure local full coverage between such campaigns, even in a rolling campaign scenario (Figure 8). On a five-year campaign cycle usage rates would fall to under 50% between campaigns. A three-year cycle combined with the TNVS could potentially maintain universal coverage locally throughout the period, although this strategy results in the delivery of approximately 20 million excess LLINs, due to oversupply in the years that the campaigns are implemented.

Over 10 years this combination would require 63.3 million LLINs at a total cost of $444 million, and would provide 82% of total person-years of protection, at a cost per person-year protected of $1.35, and a per-net cost of $7.01.

#### Combination 2: TNVS plus school voucher

**Figure 9:**
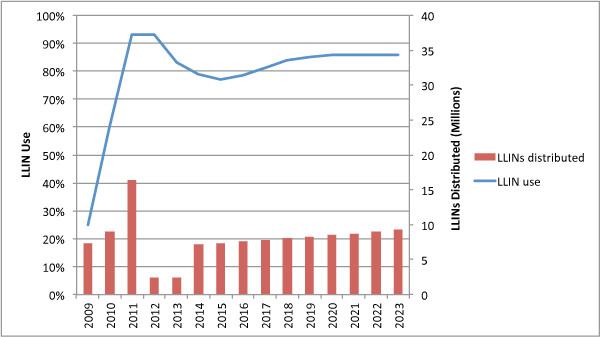
TNVS plus limited school voucher.

Figure 9 illustrates the combination of the TNVS with a school voucher distributed each year in Standards 1, 3, 5, 7 and Forms 1 and 4. Distributing nets to all primary school students would result in an oversupply of nets; distributing nets to students every second year achieves the overall goal but assumes that within households nets will be shared among family members not of school age, and allow for replacement of the oldest nets in the household as the student moves through school. Targeting pregnant women and infants through the TNVS, and students in these school grades leads to a sustained coverage of about 82%. Beneficiary identification is straightforward for reasons mentioned earlier. The use of vouchers for schools consolidates administration of the programme into one activity, rather than working with multiple mechanisms in which vouchers are combined with a separate free net distribution. Over 10 years the combination of TNVS plus a school voucher would require 65.4 million LLINs at a total cost of $449 million, and would provide 82% of total person-years of protection, at a cost per person-year-protected of $1.34, and a per-net cost of $6.87.

#### Combination 3: TNVS plus school voucher plus community voucher

**Figure 10:**
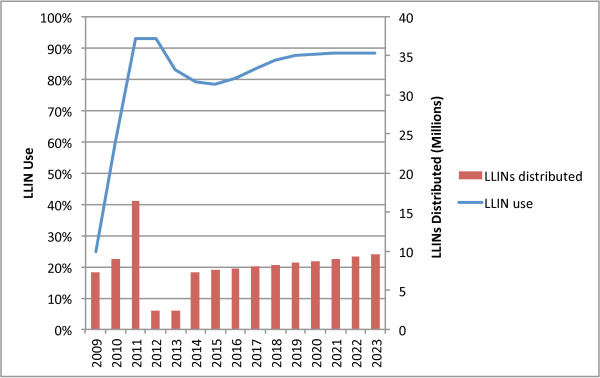
TNVS plus limited school voucher plus limited community voucher.

Figure 10 illustrates the combination of the TNVS, a more limited school voucher (given to Standards 1, 4, 7 annually) and a limited community voucher (to supply two million LLINs in 2014). In modelling this approach the TNVS plus the limited school voucher would still require substantial input to maintain universal coverage as defined by NMCP, and the community voucher has been scaled to meet the remaining need. Criteria for identifying the households would need to be developed. One possible avenue for such would be to target the remaining ~16% of households in Tanzania that have neither a pregnant woman nor any children under 15 years of age. Over 10 years this combination would require 69 million LLINs at a total cost of $520 million, and would provide 85% of total person-years of protection, at a cost per person-year-protected of $1.52, and a per-net cost of $7.54.

Summary modelling results are included in the Additional file 2.

## Discussion

### Community delivery of nets

The most significant difficulty presented by the community-based distribution approach is how to define and identify households that qualify for a new net or voucher in an equitable and implementable manner. Criteria for identifying these households would need to be transparent and consistently applied; reporting and supervision would likely need to be intensive in order to meet donor reporting requirements showing that the system is reaching the right households. At the same time, communities should have some leeway in determining the most needy households. Verification that new nets are needed at the household level may be difficult; an alternative is to institute a net replacement programme where worn nets are exchanged for new ones. However, such a system might reward households that wear their nets out more quickly.

Community-based distribution could provide a more continuous distribution mechanism but likely at a slightly higher cost per net delivered, due to smaller and more frequent deliveries to village level (if direct distribution is used), and an expansion of the monitoring system being required. Semi-annual or quarterly net distribution is recommended for direct deliveries or continuous voucher availability. A voucher, particularly if the top-up is increased slightly, may provide a limiting factor to reduce the number of households that obtain more nets than they need. A steady supply of vouchers would need to be issued over the course of the year with caps at a village level to avoid rapid depletion of capital from the programme for voucher redemption (this could be estimated based on UCC registration numbers, but growth rates for specific village/ward levels may be highly variant due to dramatic differences in migration and urbanization rates; registries would need to be updated regularly to ensure adequate supply volumes if nets rather than vouchers are delivered to households). A retailer scale-up programme would also be necessary if a voucher mechanism is used. To ensure that criteria for voucher or net allocation are met at the village level a significant investment in the supervision system will be necessary.

### School-based distribution

Collaborations between the health and education system are not new; health staff within the school system have also implemented Vitamin A, mebendazole and praziquantel campaigns. Collaboration could be coordinated through District School Health Coordinators and School Health Committees as with these other campaigns. Logistically, vouchers would be easier to distribute than nets, and this system would enable the retail networks to expand stocks, potentially making this a more attractive option as volumes of net sales increase, as well as to allow options for net choice which might not be possible with direct LLIN distribution through schools. Distribution of free nets at schools could be done all at once but would require significant storage and management logistics. Any school-based distribution option must take into consideration the logistical burden on school staff.

The timing of distribution is also important. Distributing large numbers of vouchers at the beginning of the school year would mean that retailers would require large stocks of nets at one time. It may be preferable to distribute vouchers to school children more or less evenly throughout the year, perhaps by grade level, in order for retailers to maintain a steady level of stock, as they may be unable to scale up to meet demand by all students at once.

**Figure 11:**
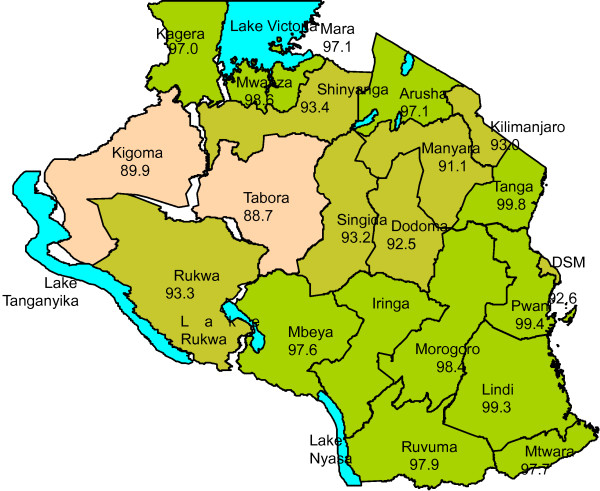
Primary school enrolment by region (2010).

Retailers have largely set up around the clinics to capture the maximum number of vouchers as they are issued. As the current network is rather saturated, having the schools as an additional voucher distributing point creates the possibility to bring in new retailers who can situate themselves close to schools. However, the challenge will be to have them in place at the start of implementation of the strategy, as few may be willing to take the risk of investment before seeing the vouchers actually distributed. Additionally, consideration of school attendance and enrolment rates, the latter of which are high in Tanzania (Figure 11), should take into account the fraction of children that attend boarding schools and thus would return home irregularly and thus not deliver nets the household. This was not considered in the estimates included in the paper and this is a potential limitation of this research.

### Commercial sector

While Tanzania previously had multiple brands of locally made ITNs bundled with insecticide treatment kits available at both full retail and subsidized prices, the NMCP switched to LLINs in 2009, and Tanzania ceased importation of retreatment kits by 2010. Textile Manufacturers of Tanzania Limited (TMTL) and Moshi Textile Mills Limited (Motex) ceased production of untreated polyester nets in July 2009 and March 2010 respectively. The remaining Tanzanian net manufacturers, A-Z and Sunflag, were expected to run out of bundled polyester nets due to lack of available insecticide retreatment kits by September 2011 [33].

It is not clear whether there is a significant market for unsubsidized LLINs outside urban areas. Internationally recognized, WHOPES-recommended LLINs (manufactured by A-Z, BASF, Vestergaard Frandsen and BestNet) are available on the Tanzanian commercial market in small quantities but these remain an insignificant portion of the LLINs available in country.

Due to the highly limited information on demand at various prices, it is difficult to predict the coverage that would result from a manufacturer subsidy. However, evidence suggests that such a system would not be equitable except at an extremely low retail price, and retailers are unlikely to accept a recommended retail price of TZS500 given their current dissatisfaction with this margin under the TNVS [10]. Current information on the equity of the TZS500 voucher is not yet available. Even if nets are highly subsidized at manufacturer level, as nets move through the retail chain, prices are likely to rise to a level beyond the ability to pay for a large proportion of Tanzanian households.

### Combinations

Many of the proposed individual mechanisms for LLIN “Keep-Up” will likely fail to deliver adequate numbers of LLINs to maintain universal coverage. Combination approaches to net delivery have been tried previously in some settings including PSI’s “Malawi model” that combined traditional social marketing with highly subsidized nets delivered to pregnant women and children under five through ANC-EPI clinics in Malawi [34,35]. Kenya’s mix of ANC distribution, subsidized nets and campaigns has been described elsewhere [26,36-39]. In the Tanzanian “Keep-Up” context combinations of approaches may allow the achievement of LLIN delivery on the scale needed to maintain universal coverage and at the same time allow for the targeting of different households based on the targeting criteria of each selected channel.

Special attention needs to be paid to the overlap of households targeted by various channels in combined approaches. NetCALC assumes inter-household reallocation and thus approaches that focus on specific target groups may appear in the model to increase population level coverage even though in reality nets would concentrate within households in the absence of such reallocation. Some combinations such as TNVS plus primary school vouchers may deliver nets to the same household and as such NetCALC may overestimate coverage results based on the overlap. This targeting of households through multiple channels may be seen as both an advantage and disadvantage of using combined approaches depending on the demographics of the country and the various channels chosen in combination.

### TNVS plus rolling campaigns

Tanzania has already combined universal campaigns with ongoing TNVS; preliminary evidence (MEDA, pers comm) indicates that the mass distribution campaigns did not significantly dampen the demand for voucher redemption, despite predictions to the contrary, indicating that it may be possible to combine free LLIN delivery with a subsidized system in this context. In this model, national coverage remains high and the costs of this strategy are comparatively low, though local coverage levels will still fluctuate greatly between distributions.

A continuous rolling campaign by zone would require a dedicated campaign team. This would be preferable to adding campaign planning to the existing workload of already busy NMCP and implementing partner staff.

Mass campaigns need to remain a tool in the arsenal of the Tanzania NMCP, but are better suited for emergency “catch-up” situations when national or sub-national areas have fallen below target levels of ownership and use of LLINs and will not recover using the country’s “Keep-Up” strategy; during epidemics or emergency situations, or alternatively for the regular coverage of institutional sleeping spaces such as boarding schools, prisons, hospitals and military camps.

### TNVS plus school-based distribution

**Figure 12:**
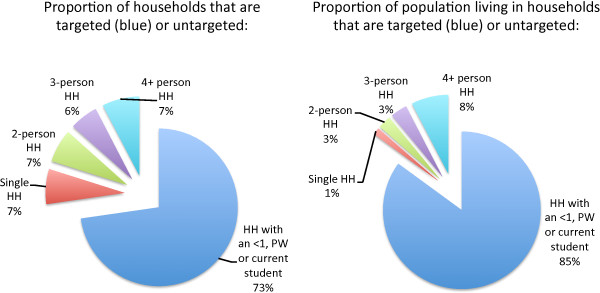
**Proportion of targeted and untargeted households and population living in them.** The school voucher + TNVS approach would reach the 73% of households (left) that have at least one member of the target groups. Because the untargeted households are smaller in size than targeted households, a greater proportion of the population (85%) is reached through this combination.

The TNVS plus a school-based method of delivery has the potential to reach a large majority of households, shown in Figure 12. Approximately 73% of households, representing 85% of the population, include a currently pregnant woman, an infant (under one year) or a current student [18]. The remaining untargeted households are split evenly among single-person households (7% of all households and 1% of the population), two-person households (7% of all households and 3% of the population), three-person households (6% of all households and 3% of the population), and households with four or more people (8% of all households and 8% of the population). Of the single-person households, roughly two-thirds (65%) are male. Of the two-person households, 73% are male-female households, split 44/56 between couples under and over age 40. Roughly two-thirds of the larger untargeted households have one to three children between the ages of one and five, and thus would have recently been eligible for the TNVS (both the pregnant woman and the infant voucher). These households would be expected to move into the targeted category as the children reach school age.

The combination of the TNVS plus school voucher distribution is the only option with a clear and straightforward identification mechanism for beneficiary households that could also supply nets on a continuous basis, avoiding significant drops in coverage. While scaling up retailer stocks to meet the increased demand would require significant initial seed investment, and the logistics of voucher distribution through the school system remain to be developed in detail, the advantages of this option make it a promising one for reaching a large proportion of households within Tanzania with replacement nets.

### TNVS plus community-based distribution

TNVS plus a community distribution has the potential to reach 100% of households – but given geographic barriers in remote areas, bias favouring households that are more central to the distribution site or voucher holder, political considerations, and the good but varied success of the UCC and hang-up campaigns at reaching all households with the correct number of nets, 100% coverage of all households is not guaranteed under such a mechanism.

### Choice

While the majority of stakeholders expressed desire to have a range of choices available for nets (for size, shape, material and colour), incorporating choice of nets within the retail sector on multiple parameters would likely require massive working capital injections in order to ensure availability of a variety of options at each retail location. Vouchers do offer an obvious mechanism for incorporation of choice compared with direct net delivery, and this process is simplified when the voucher represents a fixed value and the consumer provides a top-up that corresponds with the difference in price of their preferred net.

### Scaling up LLIN stocks at retailer level

Initial seed funding from the President’s Malaria Initiative (PMI) and A-Z was used in 2010-11 to increase working stocks of nets for around 3,000 retailers, using a “buy five get 10 free” programme, in which PMI and A-Z each provided five free nets to retailers who purchased five themselves. This fund has been exhausted and the newer and smaller retailers interviewed found it difficult to finance scale-up due to shortage of working capital. Even though a large number of retailers reported working capital as their utmost constraint for business expansion, a close analysis of their business showed that they lack basic business management skills like record keeping, stock planning, cash management, etc, that are crucial for managing their businesses. There is, therefore, a need to provide training to retailers in addition to any potential credit mechanism.

If school or community vouchers are issued only once or twice per year, retailers would need significant capital in order to scale up stock levels to meet the large demand for nets immediately following the voucher distribution. If voucher distribution is more evenly spaced throughout the year, demand is likely to be lower on average and more consistent, and retailers would require a smaller amount of capital in order to scale up stocks of LLINs to meet demand.

### Behaviour change communication and net durability

Net durability is comprised of the bio-availability of insecticide on the net over time, the net’s physical condition (holes, tears, burns, etc), and perhaps most importantly, the perception of the user about their net’s usefulness. BCC was cited in every stakeholder meeting as a key component of any “Keep-Up” strategy, to stimulate demand for nets and encourage consistent use throughout the year. Along with improvements in the physical durability of LLINs, “care and repair” messaging also has the potential in theory to increase the useful life of nets, although by how many months remains to be quantified. Should LLIN effective lifetimes be substantially longer than under the base scenarios, net replacement needs could be substantially lower. This factor is one of the largest cost drivers in the modelling exercise. If mean lifespan is two years, 89 million nets would be needed; if mean lifespan is three years, 63 million nets would be needed; a mean lifespan of four years would require 51 million nets over 10 years. Textile improvements and BCC activities that could significantly extend the life of LLINs should be explored and evaluated.

### Population estimates

Experience from the recent mass campaigns indicates that Tanzania’s 2002 Census figures and subsequent projections by the National Bureau of Statistics may be underestimating the population by as much as 15% [17]. This represents a significant difference with implications for procurement and planning. In the estimates presented here NBS population projections are used, but planning committees should consider including room for this level of population variance in final budgets.

## Conclusions

The “Keep-Up” strategy that is most cost-efficient to maintain universal coverage will be the strategy that best optimizes the numbers of nets needed over time, while maintaining universal coverage of LLINs. Based on the modelling and costing shown here, in combination with the qualities of a desirable system identified by stakeholders, it appears that a TNVS targeting pregnant women and infants plus school-based voucher distribution (to Standards 1, 3, 5, 7, and Forms 1 and 4) is the best strategic option. The strategy has a very clear and simple identification strategy, the ability to reach 85% of the population when combined with the TNVS, and could be clearly monitored. A modified version of this approach whereby physical nets are distributed to these students, rather than vouchers, is now being piloted in 20 districts in the country to assess field effectiveness, and to examine in particular whether targeted households redistribute excess nets to untargeted households. Repeated mass campaigns and some level of private sector delivery remain strong potential back-up strategies in the event of failure of the selected “Keep-Up” strategies.

## Abbreviations

ANC/EPI: Ante natal care/expanded programme on immunization; A-Z: A-Z Textile mills Ltd; BCC: Behaviour change communication; ITN: Insecticide-treated net; LLIN: Long-lasting insecticidal net; MEDA: Mennonite economic development associates; MSD: Medical stores department; Motex: Moshi textile manufacturing limited; NBS: National bureau of statistics; NMCP: National malaria control programme; PMI: President’s malaria initiative; PSI: Population services international; RBM: Roll back malaria; RCH: Reproductive and child health; SMARTNET: Strategic social marketing for expanding the commercial market of ITNs in Tanzania; Swiss TPH: Swiss tropical and public health institute; TNVS: Tanzania national voucher scheme; TMTL: Textile manufacturers of tanzania limited; TZS: Tanzanian shilling; U5CC: Under-five catch up campaign; UCC: Universal coverage campaign; USAID: United States agency for international development; USD: United states dollar; VCWG: Vector control working group; WHO: World health organization.

## Competing interests

The authors declare that they have no competing interests. At the time of the fieldwork, Nick Brown was head of the ITN Cell at the Tanzania NMCP. He is currently employed by A-Z Textile Mills Ltd.

## Authors’ contributions

HMK led the fieldwork and drafted the manuscript; JOY conducted the modelling and fieldwork and contributed to the manuscript; AM conducted fieldwork and contributed to the manuscript. RM, NB and CL contributed to the study design. AK designed the NetCALC tool. All authors read, edited and approved the final manuscript.

## Supplementary Material

Additional file 1: Tables S1 Interviews and stakeholder participation, by location. Additional file 1: Table S1 summarizes the number and types of stakeholders consulted through meetings and interviews during the fieldwork, by location. Click here for file

Additional file 2: Tables S1 Summary results of LLIN coverage and usage modelling over period 2012-2021. Additional file 2: Table S2 summarizes results of NetCALC modelling over the period of 2012-2021 for various options for maintaining universal coverage. All options mentioned in the paper are presented here and described in % of person-years-of-protection (PYP) obtained, number of LLINs required, total cost in USD, cost per LLIN, cost per PYP, and number of excess LLIN delivered for each option. Click here for file
