# Supplementary material for: Analysing and recommending options for maintaining universal coverage with long-lasting insecticidal nets: the case of Tanzania in 2011
Source: Malar J. 2013 May 4;12:150. doi: 10.1186/1475-2875-12-150 (PMC3694474; doi:10.1186/1475-2875-12-150)
Supplement: Additional file 1: Tables S1 — Interviews and stakeholder participation, by location. Additional file 1: Table S1 summarizes the number and types of stakeholders consulted through meetings and interviews during the fieldwork, by location. [file 1475-2875-12-150-S1.pdf]

Interviews and stakeholder meeting participation

	Dar es Salaam	Morogoro	Mtwara	Mwanza	Arusha	Dar es Salaam (second meeting)	Dar es Salaam Final	**Total**
Number of regions participating in zonal stakeholder meetings	1	3	3	7	4	1	8	**18**
Number of participants[o] at the zonal stakeholder meeting	36	~35[a]	~35[a]	46	47	26*	41	**225**
Number of district field visits		1	1	2	2	3*		**9**
Number of RCH clinics visited		1	1	2	2			**6**
Number of retailers interviewed		2	3	2	2	2*		**11**
Number of net manufacturers interviewed	1	1	0	1	3			**6**
Number of CBOs and Tanzanian NGOs interviewed	2	0	1	2				**5**
Donor agencies interviewed	5							**5**
International NGOs interviewed	6							**6**

[o] Participants included regional and district medical officers, reproductive and child health clinic nurses, regional and district malaria focal persons, council health management team members, district executive directors, regional administrative secretaries, municipal executive officers, village executive officers, principal health officers, retailers, community members, implementing partner staff, national malaria control programme staff, net manufacturers and distributors, and in Dar es Salaam, implementing partner staff, donor agencies and other NGOs.

[a] Attendance sheets were lost and exact numbers are not known.

* Field visits were not conducted but district municipal council members and retailers from Temeke, Kinondoni, and Ilala were consulted during a separate stakeholders meeting.
