# Supplementary material for: Analysing and recommending options for maintaining universal coverage with long-lasting insecticidal nets: the case of Tanzania in 2011
Source: Malar J. 2013 May 4;12:150. doi: 10.1186/1475-2875-12-150 (PMC3694474; doi:10.1186/1475-2875-12-150)
Supplement: Additional file 2: Tables S1 — Summary results of LLIN coverage and usage modelling over period 2012-2021. Additional file 2: Table S2 summarizes results of NetCALC modelling over the period of 2012-2021 for various options for maintaining universal coverage. All options mentioned in the paper are presented here and described in % of person-years-of-protection (PYP) obtained, number of LLINs required, total cost in USD, cost per LLIN, cost per PYP, and number of excess LLIN delivered for each option. [file 1475-2875-12-150-S2.pdf]

Summary results of LLIN coverage and usage modelling over period 2012-2021. All costs in 2011 USD.

Distribution System	% of person-years protected (PYP) 2012-2021	# LLIN delivered (millions)	Total Cost (million USD)	Cost per LLIN[1]	Cost per PYP	Excess LLIN (millions)[2]
**Tanzania National Voucher Scheme (TNVS)/Antenatal Care-Expanded Programme on Immunization (ANC-EPI)-based approaches**						
TNVS only	45%	26.5	$182	$6.87	$0.99	2.7
ANC-EPI net distribution	48%	29.5	$212	$7.19	$1.10	2.9
**Universal Coverage Campaign (UCC)-based approaches**						
UCC (3 year cycle)	77%	61.5	$406	$6.59	$1.32	11.6
UCC (5 year cycle)	54%	47.0	$249	$5.30	$1.18	2.7
**Community-based approaches**						
Community voucher	78%	62.7	$477	$7.61	$1.52	2.7
Community nets	78%	62.7	$432	$6.89	$1.39	2.7
Household net card/voucher (1 per	78%	62.4	$475	$7.61	$1.52	2.7

Distribution System	% of person-years protected (PYP) 2012-2021	# LLIN delivered (millions)	Total Cost (million USD)	Cost per LLIN[1]	Cost per PYP	Excess LLIN (millions)[2]
household per year)						
**School-based approaches**						
Large Scale School[3] Voucher	73%	56.7	$390	$6.87	$1.32	2.7
Large Scale School Nets	83%	67.4	$466	$7.00	$1.41	2.7
**Commercial sector-based approaches**						
Subsidized sales[4] TZS500	52%	33.4	$214	$6.40	$1.00	2.7
Subsidized sales TZS1,000	49%	30.3	$188	$6.18	$0.93	2.7
Mixed Top Up Sales (mixed subsidized sales)	52%	33.4	$209	$6.26	$0.97	2.7
**Combination approaches**						
**Two-way combinations**						

Distribution System	% of person-years protected (PYP) 2012-2021	# LLIN delivered (millions)	Total Cost (million USD)	Cost per LLIN[1]	Cost per PYP	Excess LLIN (millions)[2]
UCC (3-year cycle) + TNVS	82%	63.3	$444	7.01	$1.35	6.2
UCC (5-year cycle) + TNVS	75%	71.1	$432	$6.06	$1.45	12.9
TNVS + Limited School Voucher	82%	65.4	$449	$6.87	$1.34	2.7
TNVS + Large School Voucher	93%	86.9	$597	$6.87	$1.57	37.0
Commercial (subsidized) + TNVS	76%	58.0	$386	$6.65	$1.23	3.2
Commercial (subsidized) + School (Limited)	77%	58.8	$391	$6.65	$1.24	0.2
Commercial (subsidized) + Community Voucher (limited)	86%	68.2	$453	$6.64	$1.29	4.2
**Three-way combinations**						
TNVS + School Voucher (Limited)+	85%	69.0	$520	$7.54	$1.52	2.7

**Distribution System**	**% of person-years protected (PYP) 2012-2021**	**# LLIN delivered (millions)**	**Total Cost (million USD)**	**Cost per LLIN[1]**	**Cost per PYP**	**Excess LLIN (millions)[2]**
Community voucher (limited)						
Commercial (subsidized) + TNVS + School (limited)	93%	85.8	$577	$6.72	$1.51	33.5
**Four-way combinations**						
Commercial (subsidized) + TNVS + School (limited) + Community (limited)	93%	111.5	$799	$7.17	$2.12	106.1

1. Cost per LLIN is the average cost per LLIN delivered over the period 2012-2021
2. Excess LLIN = the number of LLIN delivered in excess of need for full coverage and is calculated on an annual basis, the number shown refers to the entire period 2012-2021
3. School = School voucher unless otherwise noted (limited refers to vouchers given only to certain classes while large scale refers to delivery to all primary school students
4. Commercial (subsidized) = Manufacturer Subsidy
